# Gait Patterns in Hemiplegic Patients with Equinus Foot Deformity

**DOI:** 10.1155/2014/939316

**Published:** 2014-04-22

**Authors:** M. Manca, G. Ferraresi, M. Cosma, L. Cavazzuti, M. Morelli, M. G. Benedetti

**Affiliations:** ^1^Movement Analysis Laboratory, Neuroscience and Rehabilitation Department, Via della Fiera, 44124 Ferrara, Italy; ^2^Physical Therapy and Rehabilitation Unit, Istituto Ortopedico Rizzoli, Via Pupilli 1, 40136 Bologna, Italy

## Abstract

Equinus deformity of the foot is a common feature of hemiplegia, which impairs the gait pattern of patients. The aim of the present study was to explore the role of ankle-foot deformity in gait impairment. A hierarchical cluster analysis was used to classify the gait patterns of 49 chronic hemiplegic patients with equinus deformity of the foot, based on temporal-distance parameters and joint kinematic measures obtained by an innovative protocol for motion assessment in the sagittal, frontal, and transverse planes, synthesized by parametrical analysis. Cluster analysis identified five subgroups of patients with homogenous levels of dysfunction during gait. Specific joint kinematic abnormalities were found, according to the speed of progression in each cluster. Patients with faster walking were those with less ankle-foot complex impairment or with reduced range of motion of ankle-foot complex, that is with a stiff ankle-foot complex. Slow walking was typical of patients with ankle-foot complex instability (i.e., larger motion in all the planes), severe equinus and hip internal rotation pattern, and patients with hip external rotation pattern. Clustering of gait patterns in these patients is helpful for a better understanding of dysfunction during gait and delivering more targeted treatment.

## 1. Introduction

A great deal of effort has been made to develop classifications of spastic gait deviations to reduce the complexity of this disorder, improve diagnosis and clinical decision making, and facilitate communication among clinicians [1–7]. Cluster analysis has been frequently applied to detect gait patterns particularly in children with cerebral palsy [8–10]. Over the last ten years, this method has been applied to detect gait patterns or clusters not easily identifiable with standard techniques in stroke patients to classify groups of individuals with similar gait patterns [11–15]. This method has the advantage of taking into account several parameters at the same time rather than a single one for each patient. Dividing data into meaningful groups (clusters) allows capturing the main features of the gait deviation and reflecting homogenous levels of function impairment at each joint [15].

Although most studies have focused on gait clusters of stroke patients, the presence of an equinus foot was not specified; only one study [15] concentrated on equinus deformity using temporal-distance parameters and sagittal joint kinematics during gait. Equinus foot is present in fact in 10–20% of stroke survivors and is considered to be the most detrimental consequence of stroke for gait effectiveness. Appropriate knowledge of its role in stroke gait pattern can provide more targeted and effective rehabilitative treatments.

In these previous studies on cluster analysis of stroke patients, gait velocity was considered a strong determinant for group placement [11]. Furthermore, gait velocity is deemed to be a valid and reliable measure of walking recovery after stroke and has been shown to be a valuable indicator of future health and function [16, 17]. However, the amount of change that is considered to be clinically meaningful and reflective of the level of community ambulation has not been established. In a previous study [18], the present authors showed that correction of equinus foot by surgical intervention, although not determining an increase in the speed of progression, modified ankle-foot kinematics, thus explaining the gains reported by the patients and the subject-specific goals attained through treatment, such as increased stability on the foot, removal of ankle-foot orthoses, modification of shoes, walking aids, and relief of pain. With regard to this, the assessment of ankle-foot deformity during gait is relevant for appropriate clinical decision making. The attainment of other goals in gait recovery might in fact be relevant even in patients without a chance of improving their velocity. A three-dimensional kinematic approach is furthermore essential in this respect for the complete assessment of this complex deformity [18].

The aim of this study was to focus on the role of foot-ankle complex dysfunction in gait patterns in hemiplegic patients using a gait analysis protocol which allows full assessment of ankle-foot complex kinematics in the three planes of the space. A nonhierarchical cluster analysis based on temporal-distance and kinematics of the whole lower limb joints was applied for this purpose. The hypothesis that ankle-foot complex kinematics plays a major role in gait dysfunction was stated.

## 2. Material and Methods

### 2.1. Subjects

The study was carried out retrospectively on a group of subjects who were referred to the Motion Analysis Laboratory of Rehabilitation Medicine Unit in Ferrara for a routine assessment process in the period 2009-2010. Forty-nine consecutive hemiplegic patients with a stabilized clinical condition (30 men and 19 women, 22 with left hemiplegia and 27 with right hemiplegia) and equinus deformity of the foot were enrolled in the study by an experienced clinician (M.M.). The inclusion criteria were hemiplegia at least six months before enrolment in the study, equinus deformity evaluated at the observational analysis and clinical assessment, ability to walk independently for at least 10 m, and absence of previous orthopaedic surgery. Patients were excluded if they had received botulinum injections or a phenol nerve block in the hemiplegic lower limb 6 months before the evaluation and if they had other medical disorders which might have adversely affected their gait pattern.

### 2.2. Procedures

Gait analysis was performed on a 10 meter walkway with patients walking at a self-selected speed, barefoot, and with or without aid devices (cane).

Three-dimensional kinematics of the lower limbs and time-distance parameters were recorded using a six-camera (Mcam 50, 100 Hz) motion analysis Vicon 460 system (Vicon Oxford Metrics, Oxford, UK). The gait analysis protocol Total3Dgait (T3D) [19], including anatomical-based description of foot joint motion in the three planes of space, already validated on stroke patients [20], was used. Three trials were collected for each subject.

### 2.3. Statistical Analysis

A set of 38 discrete parameters of amplitude were selected at crucial points of kinematic curves (maximum or minimum values in the stance and swing phase) and values at particular instants of the gait cycle (heel contact, toe-off), according to Benedetti et al. [21]. These variables were, respectively, pelvis maximum rotation, hip flexion at foot contact, hip flexion at toe-off, hip maximum extension in stance, hip maximum flexion in swing, hip total range of movement, knee flexion at foot contact, knee maximum flexion at loading response, knee maximum extension in stance, knee flexion at toe-off, knee maximum flexion in swing, knee total range of movement, ankle flexion at foot contact, ankle plantarflexion at loading response, ankle maximum dorsiflexion in stance, ankle plantarflexion at toe off, ankle maximum plantarflexion in swing, ankle range of motion in the sagittal plane, pelvis minimum rotation, hip maximum adduction in stance, hip maximum abduction in swing, hip range of motion, knee maximum adduction in stance, knee maximum abduction in swing, knee range of motion, ankle maximum abduction in stance, ankle maximum adduction in swing, ankle range of motion in the coronal plane, pelvis minimum rotation, hip maximum internal rotation in stance, hip maximum external rotation in swing, hip total range of motion, knee maximum internal rotation in stance, knee maximum external rotation in swing, knee total range of motion, ankle maximum eversion in stance, ankle maximum inversion in swing, and ankle total range of motion in transverse plane. Stance duration, stride length, cadence, and speed of progression were also included in the cluster analysis. They were normally distributed according to the Kolmogorov-Smirnov test.

As the dataset included 146 gait cycles from 49 patients, a bias due to the patient “effect” was hypothesized. For this reason, a two-step clustering analysis was considered to be reliable and stable for a dataset with continuous variables [22]. This analysis clusters the dataset into many small subclusters, which are then collected into the desired number of clusters using a hierarchical algorithm.

The number of clusters was chosen automatically using both the Akaike's information criterion (AIC) and Bayes information criterion (BIC); the first one generally overestimates and the latter one generally underestimates the number of clusters. The smaller the number is the better the model is. As they converged on the same number of clusters, we accepted that number as a reliable one. The Euclidean distance was applied as measure of distance and the variables were *z*-standardized. Post hoc analysis of the clusters, corrected for multiple comparisons by the Bonferroni method, was performed to identify the variables characterizing each cluster. Only significant variables for *P* < 0.001 were considered.

The Kruskal-Wallis test was used to explore differences with respect to age, distance from the event, and BMI of patients in the identified clusters (*P* < 0.05).

Statistical analysis was performed using SPSS v.19.0 (IBM Corp., Armonk, NY, USA).

## 3. Results

147 gait cycles of 49 patients were subdivided by AIC into homogeneous subgroups (clusters). One gait trail corresponding to one patient was excluded as an outlier. Five clusters of parameters representative of gait patterns were identified according to the clustering algorithm: cluster 1 included 34 cycles (23.3% out of the cycles analyzed), cluster 2 included 33 cycles (22.6%), cluster 3 included 26 cycles (17.8%), cluster 4 included 22 cycles (15.1%), and cluster 5 included 31 cycles (21.2%) (Table 1).

No significant difference was present among clusters in terms of age of patients, time from the event, and BMI (Table 2).

**Figure 1:**
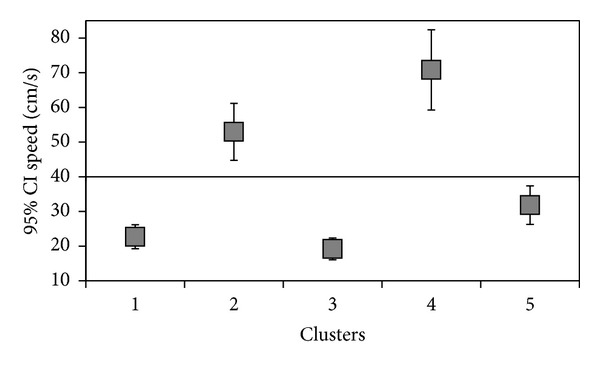
Identification of clusters with respect to speed.

The five clusters identified were different for walking speed: three clusters (1, 3, 5) were characterized by a low walking speed (resp., 34.0, 19.2, 31.8 cm/sec), one (cluster 2) by intermediate walking speed (52.9 cm/sec) and one (cluster 4) by fast walking speed (70.8 cm/sec) (Figure 1).

**Figure 2:**
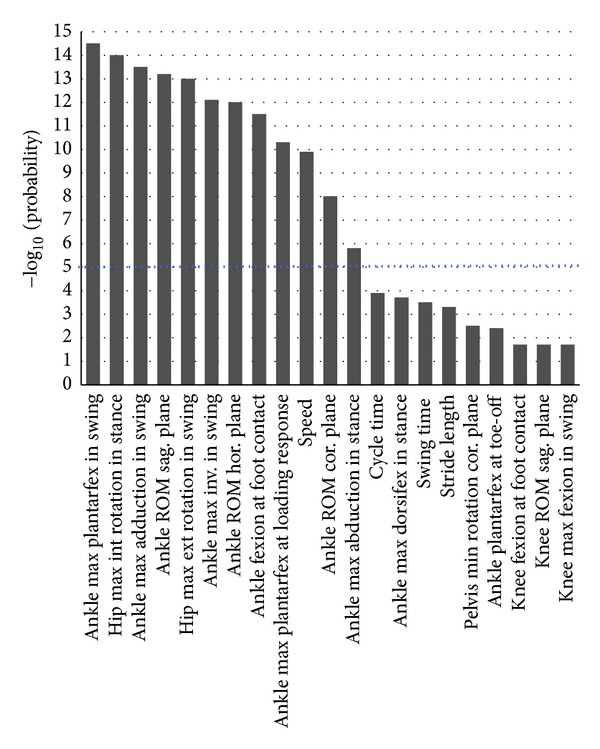
Example of clusters characterization (cluster 1). Above of the dotted line are the variables with statistical significance of *P* ≤ 0.001.

The kinematic and temporal-distance parameters characterizing the 5 clusters with statistical significance (*P* ≤ 0.001) were considered (Figure 2).

The following parameters were not considered as they did not reach statistical significance in any cluster: pelvis maximum rotation in the coronal plane, hip maximum adduction in stance, hip total range of movement in the transverse plane, knee maximum adduction in stance, knee maximum abduction in swing, knee total range of movement in the coronal and transverse planes, and knee maximum internal rotation in stance. All the ankle-foot complex variables were included in the clusters.

Cluster 1 was the most frequently observed (23.3%) and was characterized by low velocity (22.7 ± 9.9 cm/sec) and 11 kinematic variables (Table 3). Nine kinematic parameters referred to the ankle joint whilst two referred to the hip joint. In this cluster, gait pattern was characterized by the greatest plantarflexion at initial contact and loading response phase of the gait with respect to other clusters, reduced abduction through the stance phase and increased plantarflexion, adduction and external rotation of the ankle during the swing phase. The hip joint showed increased internal rotation throughout the stance phase and external rotation throughout the swing phase.

Cluster 2 was characterized by intermediate velocity (52.9 ± 23.1 cm/sec), reduced stride length and 7 kinematic variables. Five kinematic variables in the cluster were relative to the ankle and two to the knee (Table 3).

The gait pattern in this group was characterized by the largest knee flexion at initial contact and during the loading response phase, the smallest ankle swing plantarflexion compared to the other clusters, reduced plantarflexion at initial contact and during loading response phase, reduced ankle adduction in swing phase, and reduced ankle range of motion in the transverse plane.

Cluster 3 was characterized by the slowest velocity among clusters (19.2 ± 7.7 cm/sec), reduced stride length and by 9 kinematic variables. One kinematic variable was relative to the pelvis and 5 referred to the hip and 3 to the ankle (Table 3).

With respect to others, this cluster was characterized by increased pelvic anterior tilt, a reduced hip extension, reduced internal rotation during stance phase, reduced hip abduction during swing phase, and increased hip flexion at initial contact and toe-off. In this cluster, the ankle-foot complex showed the largest reduction of range of motion on the sagittal, frontal, and transverse planes and the largest shortening of stride length.

Cluster 4 was characterized by the highest velocity (70.8 ± 26.1 cm/sec), reduced stride length, cycle time, swing time and 6 kinematic variables. Three kinematic variables were relative to the hip, two to the knee, and one to the ankle joint (Table 3). The hip showed the highest range of motion (38.3 ± 8.9) among clusters, increased hip extension (−6.8 ± 5.6), and reduced hip flexion at toe-off. The knee showed the largest knee range of motion (51.5 ± 14) and hyperextension in stance phase (8.6 ± 3.2). At the ankle, the smallest external rotation among clusters was present during stance. In this cluster, the increased walking speed was associated to the greatest stride length, the shortest gait cycle time, and increased duration of the swing phase.

Cluster 5 was characterized by slow velocity (31.8 ± 15.2 cm/sec) and 9 kinematic variables. Two kinematic variables were relative to the hip, four to the knee, and three to the ankle (Table 3).

In this cluster, the minimum hip internal rotation in the stance phase and the maximum hip external rotation in swing phase were found. At the knee, hyperextension was present in stance and reduced flexion at toe-off. The ankle joint showed the lowest dorsiflexion in stance, the lowest range of motion on frontal plane, and the greatest abduction during stance phase among all the clusters.

## 4. Discussion

The gait of hemiplegic patients with equinus foot shows a wide variety of behaviour consistent with the different expressions of the impairment due to muscle spasticity, cocontraction, damaged motor control, extent of the CNS lesion, and structural changes of soft tissues [23]. The categorization of gait pattern by three-dimensional gait analysis provided useful information on the joint dysfunction underlying the gait disability. In particular, the hypothesis of the present study was that the ankle-foot complex plays a major role in gait dysfunction. This was confirmed by the fact that all the ankle selected variables in the three planes of the space were included in all the clusters, although with different statistical significance. Furthermore, clusters were characterized also by walking speed, which has been already reported to be the strongest determinant of group placement in a cluster analysis of stroke patients by Mulroy et al. [11].

According to Perry's classification [24], the patients in the present study presented a walking speed corresponding at a household ambulation (<40 cm/s, clusters 1, 3, 5) or a limited community ambulation (between 40 and 80 cm/s, clusters 2 and 4), whereas only a few patients had a walking speed useful for full community ambulation (>80 cm/s). This sample is very different from that considered by Kinsella and Moran [15], where the “fast” group of patients had a mean walking speed of 42 ± 16 cm/s. This difference of velocity in the two studies, together with the lack of references related to the coronal and transverse planes in Kinsella and Moran study [15], might affect the composition of the individual clusters, making a possible comparison difficult.

Amongst the patients examined in the present study, cluster 4, the one with the highest walking speed, showed most gait parameters very close to normative data. Hip and knee joints were involved in this cluster, showing a wide range of motion, which allowed a suitable stride length and a regular stance to swing ratio. There was only a reduced eversion of the ankle and a hyperextension of the knee, but that does not seem to have overly influenced the pattern of walking.

Patients in cluster 2 showed the second highest speed amongst clusters. In this case, the knee, and particularly the ankle, was the joints characterizing the cluster. A reduced range of motion was present at the ankle in the transverse plane, together with a reduced sagittal range of motion. The equinus at initial contact was small and, with respect to other clusters, the ankle reached a wider dorsiflexion during swing and a smaller adduction in swing phase. The reduced motion at the ankle might suggest a rigidity of the foot during stance, which might have allowed a more stable base of support with respect to patients in the other clusters, thus explaining the better speed of progression.

Clusters 1, 3, and 5 were characterized by slow speed. Cluster 1 presented the greatest deviations at the ankle and the greatest range of motion in all the planes when compared to the other clusters. This amplitude of motion represents reasonably a source of instability for the ankle that requires particular attention from the patient during the initial contact and the loading response phase, slowing the gait cycle and significantly influencing the speed. This data is confirmed by the increased duration of the cycle time that pattern 1 shows with respect to the other clusters. The hip in this cluster persists in internal rotation throughout the gait cycle, and, together with the high amount of ankle inversion during swing, configures an internal rotation spastic gait, typical of hemiplegia.

Cluster 3 represents patients with the slowest walking speed and also in this case the ankle is the most representative joint of the cluster. The peculiarity of this cluster was the greatest reduction of ankle range of motion in all the sagittal, coronal, and transverse planes. A severe equinus foot (even if included in the cluster with significance at *P* < 0.01) was present throughout the stance phase. The hip joint was characterized by high flexion attitude in the sagittal plane associated with pelvic anteversion to compensate for ankle stiffness.

Cluster 5 was mainly characterized by a pattern with external rotation of the hip, hyperextension of the knee joint in the stance phase, and reduced knee flexion during the swing phase associated with equinus-abducted foot.

As stated previously, it is very difficult to try to make comparisons with data from Kinsella and Moran study [15], the only one which took into account foot equinus deformity as responsible for gait impairment. Both characteristics of patients and gait analysis protocol used were in fact different from those of the present study. Even more difficult is the comparison with other studies where a cluster analysis on gait pattern in stroke patients was carried out [11–13]. In these studies, in fact it is never specified whether patients presented an equinus foot. The only consideration we can attempt to make is that patients with lower velocity of the third group in Kinsella and Moran study [15] were also the patients with the most abnormal gait pattern of all the subgroup, with a severe equinus foot dysfunction. A muscle weakness of dorsiflexors and plantarflexors was hypothesized to be responsible for the slow gait in this group of patients, as previously suggested also by Mulroy et al. [11]. In these clusters, the use of an ankle foot orthosis was considered helpful. Clusters identified in the present study in terms of large amount of motion, stiffness, or quite normal kinematics would be of interest, supported by clinical examination, in delineating homogenous groups for decision making with respect to prescribing orthotics, chemodenervation, surgery, or only exercise.

This is the first study which takes into account objectively, besides the hip and knee joints, kinematic deviation of the ankle-foot complex in the three planes of space that reveals information of interest for appropriate therapeutic intervention. However, a limit of this study is the lack of clinical assessment of range of motion and level of spasticity in the patients, which might have contributed to better explaining dynamic dysfunction during gait. Being a retrospective study, it was not possible to retrieve such clinical information for all the patients. Furthermore, to simplify the interpretation of the many gait parameters included in the cluster analysis only variables with significance at *P* < 0.001 were included. The analysis of other variables, which have a role in the clusters, although with less weight, might reveal other useful information.

## 5. Conclusion

The definition of homogenous multiple clusters helped us to identify speed-related patterns of walking in hemiplegic patients, with particular attention to the ankle-foot complex.

All the patterns characterized by greater reduction in speed showed an equinus deformity of the foot that (1) might be responsible for slow speed when a joint instability in all planes is present (cluster 1), (2) may consist of significantly reduced ankle range of motion in all the planes associated with hip flexion for progression (cluster 3), and (3) might be associated with externally rotated pattern at the hip, extensor pattern at the knee, and foot abduction (cluster 5). Faster patterns were instead related to the absence of severe impairment at the foot-ankle complex or to a rigid foot, with lesser involvement of the upper joints.

Future research should aim at exploring the effects of intervention in modifying the ankle-foot complex dysfunction.

## Figures and Tables

**Table 1:** Autoclustering.

Number of clusters	Akaike's information criterion (AIC)	AIC change^a^	Ratio of AIC changes^b^	Ratio of distance measures^c^
1	3873.849			
2	3598.036	−275.814	1.000	1.202
3	3393.501	−204.535	0.742	1.630
4	3325.170	−68.330	0.248	1.255
5	3300.742	−24.429	0.089	1.183

6	3302.954	2.212	−0.008	1.053
7	3312.444	9.491	−0.034	1.153
8	3340.366	27.922	−0.101	1.029
9	3371.712	31.346	−0.114	1.096
10	3413.251	41.540	−0.151	1.103
11	3464.696	51.445	−0.187	1.102
12	3525.088	60.392	−0.219	1.073
13	3591.474	66.386	−0.241	1.122
14	3666.712	75.239	−0.273	1.066
15	3746.426	79.714	−0.289	1.035

^a^The changes are from the previous number of clusters in the table.

^b^The ratios of changes are relative to the change for the two-cluster solution.

^c^The ratios of distance measures are based on the current number of clusters against the previous numbers of clusters.

**Table 2:** Data on patients.

Cluster	Mean	Confidence interval 95%	
		Lower limit	Upper limit
Age*			
1	44.1	35.5	52.6
2	40.0	21.0	59.0
3	54.2	46.0	62.3
4	53.2	41.4	65.0
5	55.1	48.0	62.3
Months from acute event**			
1	23.5	10.1	36.9
2	120.0	8.0	232.0
3	73.6	32.9	114.3
4	72.8	6.0	140.2
5	81.5	14.9	148.0
BMI***			
1	24.0	21.7	26.3
2	23.4	19.1	27.8
3	25.2	21.6	28.8
4	25.9	23.4	28.3
5	25.2	23.1	27.3

Kruskal-Wallis *P = 0.157, **P = 0.083, ***P = 0.757.

**Table 3:** Summary of gait analysis parameters characteristics of clusters.

	Cluster 1	Cluster 2	Cluster 3	Cluster 4	Cluster 5	Normative^#^
Time-distance parameters						
Speed of progression (cm/s)	22.7 ± 9.9*	52.9 ± 23.1	19.2 ± 7.7*	70.8 ± 26.1*	31.8 ± 15.2	127.8 ± 11.2
Cycle time (s)	2.7 ± 0.9	1.7 ± 0.4	2.1 ± 0.7	1.4 ± 0.3*	2.1 ± 0.8	1.1 ± 0.1
Stride length (cm)	55.9 ± 14.1	83.1 ± 17.6*	38.6 ± 14.6*	95.1 ± 18.4*	57.5 ± 15.6	141.2 ± 8.7
Swing time (% stride)	30.2 ± 8.1	38.2 ± 4.5	31.3 ± 7.4	41.7 ± 3.7*	39.8 ± 9.3	39.7 ± 1.7

Kinematic variables (°)						
PELVIS max rotation—Sagittal plane	21.1 ± 8.8	18.2 ± 4.1	23.1 ± 3.1*	15.5 ± 4.7	19.9 ± 4.5	10.3 ± 4.4
PELVIS min rotation—Coronal plane	5.5 ± 2.7	1.7 ± 5.0	6.3 ± 2.6	2.8 ± 3.3	3.9 ± 2.7	−3.9 ± 1.9
PELVIS min rotation—horizontal plane	−19.8 ± 7.9	−13.9 ± 7.4	−24.4 ± 15.0	−10.7 ± 9.3	−21.6 ± 10.9	−5.8 ± 2.5
HIP flexion at initial contact	23.5 ± 9.6	27.3 ± 4.5	35.8 ± 6.4*	23.1 ± 7.1	21.8 ± 4.7	30.9 ± 5.9
HIP flexion at toe-off	15.2 ± 12.5	12.5 ± 6.0	31.2 ± 7.6*	3.6 ± 8.1*	16.9 ± 15.5	−3.1 ± 6.1
HIP max extension in stance	4.35 ± 9.91	4.3 ± 6.3	23.5 ± 6.0*	−6.8 ± 5.6*	7.7 ± 4.1	−9.4 ± 5.7
HIP max flexion in swing	30.90 ± 9.78	30.4 ± 5.1	40.8 ± 9.8	31.4 ± 10.7	29.4 ± 6.1	32.6 ± 5.4
HIP max abduction in swing	−2.8 ± 4.5	−4.9 ± 3.9	1.1 ± 2.4*	−3.4 ± 3.1	−5.3 ± 3.7	−6.6 ± 2.7
HIP max internal rotation in stance	13.0 ± 6.1*	3.6 ± 12.1	−9.2 ± 6.3*	1.2 ± 9.3	−14.2 ± 13.8*	5.5 ± 9.2
HIP max external rotation in swing	8.3 ± 6.6*	−1.4 ± 11.8	−13.4 ± 9.0	−4.5 ± 8.9	−17.8 ± 12.9*	−1.3 ± 9.5
HIP ROM—Coronal plane	9.4 ± 2.9	9.0 ± 4.1	6.4 ± 2.7	12.5 ± 4.1	8.3 ± 3.9	13.7 ± 3.2
HIP ROM—Sagittal plane	27.5 ± 8.3	26.8 ± 7.2	17.8 ± 8.7	38.3 ± 8.9*	38.3 ± 8.9	42.5 ± 4.1
KNEE flexion at initial contact	6.3 ± 7.3	15.9 ± 4.9*	15.9 ± 7.9	7.0 ± 6.6	7.0 ± 6.7*	4.5 ± 3.5
KNEE max flexion at loading response	11.1 ± 10.5	19.4 ± 5.4*	16.6 ± 8.1	7.4 ± 6.7	1.8 ± 5.2*	16.0 ± 5.9
KNEE max extension in stance	−1.2 ± 11.6	5.7 ± 9.9	1.5 ± 6.5	−8.6 ± 3.2*	−9.0 ± 5.9*	4.3 ± 3.8
KNEE flexion at toe-off	18.8 ± 10.2	28.1 ± 11.0	20.3 ± 7.0	22.5 ± 8.3	11.7 ± 7.4*	38.9 ± 6.2
KNEE max flexion in swing	23.1 ± 13.5	33.4 ± 12.0	27.8 ± 8.5	42.8 ± 14.9	19.4 ± 9.9	64.9 ± 5.6
KNEE ROM-Sagittal plane	27.0 ± 11.3	29.7 ± 9.9	26.3 ± 10.5	51.5 ± 14.0*	28.6 ± 10.0	64.8 ± 4.7
ANKLE flexion at initial contact	−26.7 ± 4.4*	−9.0 ± 7.3*	−17.5 ± 13.0	−17.1 ± 5.1	−22.4 ± 4.8	2.6 ± 4.2
ANKLE max plantarflexion at loading response	−26.9 ± 4.5*	−9.8 ± 6.2*	−18.4 ± 13.6	−17.3 ± 4.9	−24.5 ± 6.2	−2.6 ± 3.9
ANKLE max dorsiflexion in stance	4.7 ± 6.7	7.1 ± 8.3	−7.4 ± 11.7	3.1 ± 6.7	−9.8 ± 7.4*	15.9 ± 4.1
ANKLE plantarflexion at toe-off	−18.8 ± 9.3	−6.6 ± 8.1	−14.2 ± 11.0	−14.3 ± 10.7	−16.3 ± 6.9	−10.4 ± 5.4
ANKLE max dorsiflexion in swing	−34.3 ± 4.6*	−11.7 ± 8.0*	−19.5 ± 12.6	−23.4 ± 9.6	−26.2 ± 10.7	−13.4 ± 6.0
ANKLE max abduction in stance	1.3 ± 5.1*	2.5 ± 6.6	11.2 ± 8.6	0.8 ± 5.4	16.0 ± 9.1*	−0.1 ± 4.5
ANKLE max adduction in swing	36.4 ± 5.1*	16.2 ± 8.8*	21.1 ± 10.8	26.5 ± 6.8	26.2 ± 10.7	20.7 ± 6.2
ANKLE max eversion in stance	7.3 ± 6.4	5.3 ± 5.7	12.2 ± 10.5	3.0 ± 3.8*	18.5 ± 10.0	3.1 ± 5.2
ANKLE max inversion in swing	40.1 ± 5.3*	26.7 ± 9.5	23.7 ± 12.2	25.7 ± 7.6	25.7 ± 7.6	13.9 ± 6.4
ANKLE ROM—Coronal plane	32.7 ± 8.8*	21.9 ± 6.9	13.4 ± 6.4*	22.7 ± 6.4	13.6 ± 6.4*	13.8 ± 2.9
ANKLE ROM—Sagittal plane	39.1 ± 7.1*	19.8 ± 5.3	14.2 ± 7.5*	27.2 ± 4.7	19.3 ± 11.3	29.4 ± 5.1
ANKLE ROM—Horizontal plane	35.3 ± 7.3*	14.8 ± 5.8*	12.8 ± 6.4*	25.9 ± 7.5	16.5 ± 7.9	21.8 ± 4.2

*P < 0.001.

^#^Normative values are relative to 20 healthy subjects (11 men and 9 women. mean age of 27.9 years. unpublished).
